# Geraniol and Carvacrol in Essential Oil Bearing *Thymus pulegioides*: Distribution in Natural Habitats and Phytotoxic Effect

**DOI:** 10.3390/molecules27030986

**Published:** 2022-02-01

**Authors:** Kristina Ložienė, Vaida Vaičiulytė

**Affiliations:** Nature Research Centre, Institute of Botany, Žaliųjų Ežerų Str. 47, LT-08406 Vilnius, Lithuania; vaida.vaiciulyte@gamtc.lt

**Keywords:** *Thymus pulegioides*, chemotypes, distribution, carvacrol, geraniol, phytotoxity, *Hypericum perforatum*, *Phleum pratense*

## Abstract

Phenolic and non-phenolic chemotypes of *Thymus pulegioides* L. are common in Europe. Essential oils of these chemotypes, as various compositions of allelochemicals, can have different phytotoxic effects on neighboring plants in natural habitats. The aim of this study was to establish the distribution of carvacrol and geraniol in *T. pulegioides*, growing wild in Lithuania, and compare phytotoxity of essential oils of carvacrol and geraniol chemotypes on selected plant species. In investigating 131 *T. pulegioides* habitats, essential oils were isolated by hydrodistillation and analyzed by GC-FID and GC-MS. Phytotoxity of essential oils extracted from carvacrol and geraniol chemotypes transmitted through water and air to selected plants was determined under laboratory conditions. Pharmacologically valuable *Hypericum perforatum* L. and the important forage grass *Phleum pratense* L. were respectively selected for experimentation from among 35 medicinal plants and 10 feed Poaceae species, growing in *T. pulegioides* habitats. Field results showed that carvacrol is common throughout Lithuania’s territory, whereas the geraniol is predominantly located under the continental climatic conditions of the eastern region of the country. In the laboratory experiment, it was established that there was stronger inhibition of *P. pratense* seed germination by the essential oil of the geraniol chemotype than the carvacrol chemotype. None of the *H. perforatum* seeds germinated after exposure to the essential oil of the geraniol chemotype. In general, this study builds on previous studies by providing further evidence that different *T. pulegioides* chemotypes have contrasting phytotoxic effects on neighboring plants within their natural habitats.

## 1. Introduction

Species of the genus *Thymus* (Lamiaceae) are aromatic, essential oil-bearing plants with characteristic intraspecific chemical polymorphism among chemotypes classified on the basis of the chemical composition of their essential oils [1]. This phenomenon has practical benefits because these chemotypes have different taste, antimicrobial, antioxidant, and flavoring properties which extend the application of oil extracts from *Thymus* spp. in food, pharmaceutical, and cosmetics industries [2,3,4,5].

Chemical polymorphism is a characteristic of large thyme (*Thymus pulegioides* L.) with thymol, geraniol, linalool, α-terpenyl acetate, fenchone, cis-sabinene hydrate, and most frequently carvacrol chemotypes found in Europe [6,7,8,9,10]. Previous research has demonstrated that the geraniol chemotype is also frequently found in *T. pulegioides*, growing in eastern and south-eastern regions of Lithuania and in Slovakia [10,11,12,13]. Geraniol and carvacrol are the main compounds of their eponymous geraniol and carvacrol chemotypes, respectively. They are biologically active compounds which are used in various industries, e.g., commercially important geraniol is characterized by antimicrobial (against foodborne and human pathogens), antioxidant, anti-inflammatory properties, is used in cosmetic and non-cosmetic products as an aromatic ingredient, an added as a flavoring agent in the beverage and food industries [14,15]; the monoterpenic phenol carvacrol has a wide spectrum of antimicrobial and antioxidant activity, and is therefore widely usable in food production and pharmaceutical industries [16,17].

Thymus pulegioides, growing wild in Lithuania, occurs among the plant communities of four phytocenological vegetation classes: *Molinio-Arrhenatheretea elatioris*, *Festuco-Brometea erecti*, *Trifolio-Geranietea sanguinei*, and *Koelerio-Corynephoretea canescentis* [18]. Some of these comprise productive valuable hayfields and grazing grasslands (for example, communities of vegetation class *Molinio-Arrhenatheretea elatioris*), others can be sources of pharmacologically valuable medicinal plants [19,20]. Essential oils of *T. pulegioides* chemotypes, as various compositions of allelochemicals, can have different phytotoxic effects on neighboring plants in natural habitats. Previous studies demonstrated both inhibition and stimulating effects of essential oils from the carvacrol and geraniol chemotypes of *T. pulegioides* and their main compounds on seed germination and radicle growth of economically valuable fodder grasses—dicotyledon *Trifolium pratense* L. and monocotyledon *Poa pratensis* L. [21].

The aim of this study was to establish the distribution of carvacrol and geraniolin *T. pulegioides*, growing wild in Lithuania, and to compare the phytotoxity of essential oils of carvacrol and geraniol chemotypes on one economically valuable Poaceae species and one pharmacologically valuable medicinal species growing in natural habitats where *T. pulegioides* occurs.

## 2. Results

### 2.1. Distribution of Carvacrol and Geraniol in Thymus pulegioides

**Figure 1:**
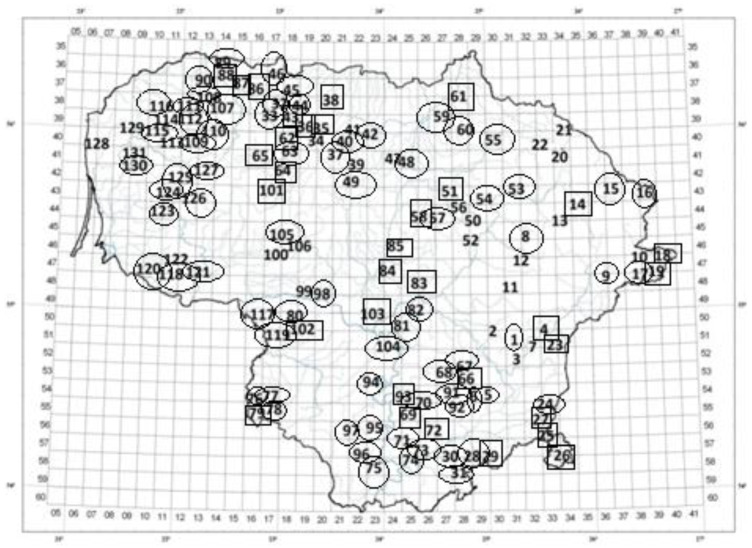
Occurrence of carvacrol and geraniol chemotype-determining compounds in *Thymus pulegioides* habitats. Habitats dominated by carvacrol or/and its precursors (*p*-cymene, γ-terpinene) are marked with a circle; those dominated by geraniol or/and biogenetically related compounds (geranial, nerol, and neral) are indicated by squares; habitats dominated by other chemotype-determining compounds (thymol, linalool, and α-terpinyl acetate) not marked in any way.

The mean percentage of cavacrol in the *T. pulegioides* habitats (N = 131) was 17.66 ± 9.33% and the mean percentage of geraniol was 6.57 ± 8.70%. Carvacrol was found in almost all habitats (except habitat no. 64) (Table 1, Figure 1); in most habitats, the percentage of this phenol varied from 5% to 25% (N = 99) and only in 14 habitats amounted to 14% or 30% higher (Table 1). Geraniol was not found in 17 of the habitats investigated. These habitats were nos. 44, 47, 59, 60, 77, 89, 94, 105, 109, 112, 115, 116, 118, 123, 125, 128, and 131. In most habitats, the percentage of geraniol was lower than 5% (more than half of the habitats); the mean percentage of geraniol was higher than 30% only in five investigated habitats (Table 1). No habitat with a dominant geraniol was found in the western part of Lithuania (Figure 1).

Four clusters of *T. pulegioides* habitats (N = 131) were distinguished by cluster analysis according to the percentages of chemotype-determining compounds and their precursors (Supplement S1). Habitats of cluster 1′ (N = 34) were distinguishable by having the largest percentage of geraniol and biogenetically related compounds (geranial, nerol, and neral) in essential oils extracted from *T. pulegioides* (Figure 1, Table 2, Supplement S1). The percentage of geraniol was 6–8 times, geranial 4–10 times, nerol 4–13 times, and neral 4–15 times higher in habitats of this cluster than in habitats of the other three clusters. The maximum percentage of geraniol was detected in cluster 1′ (habitat no. 69), but the variation in geraniol and biogenetically related compounds was the lowest here in comparison with the other clusters (Table 2, Figure 1).

Habitats of clusters 3′ and 4′ were distinguishable by the high percentage of carvacrol and its precursors *p*-cymene and γ-terpinene. In habitats of cluster 3′, the mean percentage of carvacrol was 1.5 times higher than in habitats of cluster 4′. The highest percentage of carvacrol was detected in habitat no. 31 of cluster 3′ (Figure 1, Supplement S1). The mean amount of γ-terpinene was about 6% higher in cluster 3′ in comparison with cluster 4′, while the mean amount of *p*-cymene was two times higher in cluster 4′ in comparison with cluster 3′. These two clusters encompassed more than half of investigated habitats (73 habitats) (Table 2). The thymol dominated the habitats of cluster 2′. This cluster comprised only 17 habitats. Habitats nos. 11, 34, 39, 99, 100, and 106 were not included in cluster 2′ and analyzed separately (Table 2, Supplement S1). In these habitats, linalool or α-terpinyl acetate chemotype-determining compounds, linalool or α-terpinyl acetate, respectively, dominated. These results demonstrated that carvacrol is the most frequent in the territory of Lithuania and geraniol chemotype-determining compound geraniol is less common; thymol, linalool, and α-terpinyl acetate are rare.

### 2.2. Species of Medicinal Plants and Forage Grasses of Genus Poaceae Growing in Investigated Habitats of Thymus pulegioides

The cover-abundance of *T. pulegioides* varied from + to 3 of the Braun-Blaquet scale among the different habitats. The cover-abundance of *T. pulegioides* had a value of + (occasional and less than 5% of the total 53 plot area) in 77, value 1 (abundant and with very low cover, or less abundant but with higher cover; in any case, less than 5% cover of total plot area) in 33, value 2 (very abundant and less than 5% cover, or 5–25% cover of total plot area) in 13, and value 3 (25–50% cover of total plot area, irrespective of number of individuals) in 8 habitats. Thirty-five medicinal plants, raw materials of which are included in the European Pharmacopoeia [22], were registered in the *T. pulegioides* habitats of Lithuania that were sampled (Table 3).

Four species—yarrow (*Achillea millefolium*), horsetal (*Equisetum arvense*), St. John’s wort (*Hypericum perforatum*), and agrimony (*Agrimonia eupatoria*)—were the most common and found in more than 30% of all habitats. Plants of *A. millefolium* were the most commonly detected in habitats, and in similarity with *T. pulegioides*, its cover-abundance with a value of + was found in 53% (in 70 habitats) of all habitats investigated. Only *Equisetum arvense* among these four above-mentioned species had a value of 3 in a few habitats. Although *H. perforatum* was found in more habitats than *A. eupatoria*, cover-abundance of this species of genus *Hypericum* did not exceed value + in any of the habitats.

Ten Poaceae species, characteristic of the phytocoenological vegetation classes *Molinio-Arrhenatheretea elatiori*, *Trifolio-Geranietea sanguinei*, *Koelerio-Corynephoretea,* and *Festuco-Brometea erecti*, were found in *T. pulegioides* habitats (Table 3). Cat grass (*Dactylis glomerata*) and timothy grass (*Phleum pratense*) were the most common of them and found more than in 30% of all habitats. *Dactylis glomerata* cover-abundance with a value of 1 was seven times more frequent compared with the same cover-abundance of *P. pratense*; in contrast with *P. pratense*, the highest values of *D. glomerate* cover-abundance were 3 and 4 (in habitats 3 and 1, respectively; value 4 represented range of cover-abundance from 50% to 75% in habitat).

### 2.3. Phytotoxic Effect of Essential Oils of Thymus pulegioides Carvacrol and Geraniol Chemotypes

Oxygenated monoterpenes comprised the main fraction in essential oils of vegetatively propagated clones of the *T. pulegiodes* carvacrol and geraniol chemotype—42.16% and 79.58%, respectively (Table 4 and Table 5). Carvacrol was the main chemical compound in essential oil of the carvacrol chemotype; the percentages of precursors *p*-cymene and γ-terpinene was 1.9 and 1.5 times lower, respectively, in comparison with carvacrol (Table 4). Geraniol was the main essential oil compound in the geraniol chemotype; percentages of compounds, biogenetically related with geraniol—geranial, nerol, and neral—were lower by 5.6–14.5 times in comparison with geraniol (Table 5).

The essential oils of the geraniol and carvacrol chemotypes and pure (analytical standards) geraniol and carvacrol demonstrated a strong phytotoxic effect on *P. pratense* seed germination: all germination parameters significantly differed from the control (*p* < 0.05). Essential oil of the geraniol chemotype and pure geraniol more strongly inhibited *P. pratense* seed germination than carvacrol. The strongest effect was established when seeds were affected by essential oil of the geraniol chemotype through air: only 1.3% of seeds germinated in comparison with the control. Pure geraniol also demonstrated a very strong phytotoxic effect on *P. pratense* seed germination through water: only 1.9% of seeds germinated in comparison with the control. Essential oil of geraniol chemotype had a stronger inhibitory effect through air and pure geraniol through water. Correlation of cover-abundance of *P. pratense* with geraniol percentage and combined geranial, nerol, and neral (geranial + nerol + neral) percentages in *T. pulegioides* habitats were positive but only the latter correlation was statistically significant (*r* = 0.21, *p* < 0.05). The strongest phytotoxic effect on *P. pratense* seed germination from essential oil of the carvacrol chemotype was through air (germination was 8.6 times lower in comparison with the control), and the lowest through water (germination percentage was close to control (Table 6). Cover-abundance of *P. pratense* and percentage of carvacrol in natural *T. pulegioides* habitats were not significantly correlated. The strongest inhibition effects on radicle length were from pure geraniol through water and pure carvacrol through air: radicles were 8 and 7 times lower in comparison with their respective control. It was interesting that the effects of essential oil of geraniol chemotype through air and water did not significantly inhibit *P. pratense* radicle development (Table 6).

Essential oil of the geraniol chemotype and pure geraniol strongly inhibited *H. perforatum* seed germination: not a single seed germinated. Essential oil of the carvacrol chemotype and pure carvacrol demonstrated phytotoxic effects on *H. perforatum* seed germination through air. It was interesting that pure carvacrol stimulated *H. perforatum* seed germination through water: the germination index was significantly (*p* < 0.05) higher in comparison with control (Table 7), demonstrating that more seeds germinated within a shorter time period. Correlations between cover-abundance of *H. perforatum* and percentages of carvacrol, geraniol, and geranial + nerol + neral in natural *T. pulegioides* habitats were non-significant. In all cases, the length of radicles was significantly (*p* < 0.05) lower in comparison with control. The strongest inhibition of *H. perforatum* radicle development was achieved with pure carvacrol through air: length of radicles was 3.8 times lower in comparison with the control (Table 7).

## 3. Discussion

Carvacrol is common throughout the territory of Lithuania (found in 130 habitats). Geraniol was found in 114 habitats (in 87% of habitats investigated). Thymol, linalool, and α-terpinyl acetate chemotype-determining compounds (thymol, linalool, and α terpinyl acetate, respectively) were rare. In the western region of Lithuania, the habitats were dominated by the geraniol (Figure 1). Here, the maritime climate (where temperature amplitude is lower and summers are colder than in eastern Lithuania) is more pronounced compared with elsewhere in Lithuania [25]. Published data indicate that the *Thymus vulgaris* geraniol chemotype is more resistant to low temperature and greater temperature amplitude during winter, whereas the carvacrol chemotype is more widely distributed where the winters are milder [26,27,28]. The present study showed that the mean percentage of geraniol is 2.7 times lower than cavacrol and it is more common in the essential oils of *T. pulegioides* growing in eastern Lithuania (Figure 1). Previous studies, undertaken in eastern and south-eastern Lithuania, showed that carvacrol and geraniol chemotypes of *T. pulegioides* were more common in comparison with other chemotypes, but geraniol had half the mean percentage of carvacrol [9,12,13,29,30]. Studies of the distribution of *T. pulegioides* chemotypes in other European countries, showed domination of phenolics chemotypes: carvacrol chemotype was prevalent in Romania [31,32]; the thymol chemotype in Italy [33,34]; and both thymol and carvacrol chemotypes in Portugal and Norway [35,36]. Meanwhile, the *T. pulegioides* geraniol chemotype was frequently found in Croatia [37], and geraniol and linalool chemotypes in Slovakia [8].

*Thymus pulegioides* plants, growing wild in Lithuania, were found in plant communities from four phytocoenological vegetation classes—*Molinio-Arrhenatheretea elatiori*, *Trifolio-Geranietea sanguinei*, *Koelerio-Corynephoretea,* and *Festuco-Brometea erecti* [18]. The natural and semi-natural communities of vegetation class *Molinio-Arrhenatheretea elatioris* are economically productive hayfields and grazing grasslands in Europe [19,38]. Valuable feed species of the Poaceae such as *Dactylis glomerata*, *Deschampsia cespitosa*, *Festuca pratensis*, *Phleum pratense*, *Poa pratensis,* and *Poa trivialis*, which are characteristic species of grasslands of this phytocoenological vegetation class [19]. *Trifolio-Geranietea sanguinei* grasslands which are rarely mowed because they often grow on the boundaries of meadows and forests, are floristically rich, whereas xerophytic meadows of *Festuco-Brometea erecti* and *Koelerio-Corynephoretea canescentis* are less diverse with a lower number of plant species [19,20,39]. Although grasslands of these three vegetation classes are not as valuable as *Molinio-Arrhenatheretea elatioris* hayfields and grazing grasslands, they nevertheless produce pharmacologically valuable medicinal plants which can enhance their economic value. Analysis of species composition in the habitats sampled showed that 35 medicinal species, raw materials of which are included in the European Pharmacopoeia, were registered among the *T. pulegioides* habitats (Table 3). Four species—*A. millefolium*, *E. arvense*, *H. perforatum*, and *A. eupatoria*—were the most common, being found in more than 30% of all habitats investigated. *Achillea millefolium* and *E. arvense* occur as weeds; *A. eupatoria*, although also frequent in Lithuania, is a sporadic species among plant communities of the above-mentioned phytocoenological vegetation classes. Only *H. perforatum* raw material, in contrast to *A. millefolium*, *E. arvense,* and *A. eupatoria*, is rich in pharmacologically valuable hypericins and hyperforin. According to the European Medicines Agency (EMA) assessment report published in 2009, hypericins can amount to 0.06–0.4% in herba of *H. perforatum*. Hypericins are used as an anticancer agent and a potential treatment against neurodegenerative diseases; hyperforins have antidepressant properties [40,41,42]. *H. perforatum* is a common and popular medical plant in Lithuania. It is one of characteristic species of *Trifolio-Geranietea sanguinei* vegetation class [19]. Ten Poaceae species, characteristic of *Molinio-Arrhenatheretea elatiori*, *Trifolio-Geranietea sanguinei*, *Koelerio-Corynephoretea,* and *Festuco-Brometea* phytocoenological vegetation classes, were registered in the *T. pulegioides* habitats investigated (Table 3). Only *D. glomerata* and *P. pratense* were found more than in 30 % of all investigated habitats. However, unlike *D. glomerata, P. pratense* is one of the most productive, well adapted to Northern European conditions, and has good palatability forage grass [43,44]. Therefore, *H. perforatum* and *P. pratense* were selected as experimental species for germination trials.

Essential oils can influence diversity among plant communities by affecting them phytotoxically, stimulating seed germination, and/or promoting the growth of seedlings among neighbor plants [45,46]. Monoterpenes dominant in *Thymus* essential oils can also influence plant community composition [47]. Because *T. pulegioides* is characterized by chemical polymorphism, chemotypes can differentially influence seed germination and/or seedling growth of same species. Published data indicate that thymol and carvacrol have stronger inhibitory effects than other chemotypes [48,49]. For example, *T. vulgaris* phenolic (thymol and carvacrol) chemotypes have stronger inhibition of *Bromus madritensis* [46] and *Brachypodium phoenicoides* seed germination [47] than non-phenolic chemotypes. Therefore, *T. pulegioides* carvacrol chemotype, dominant throughout the territory of Lithuania, can have various influences on the distribution of the important forage grass *P. pratense* and the pharmacologically valuable *H. perforatum*.

A significant correlational connection (*r* = 0.21, *p* < 0.05) was established only between the cover-abundance of *P. pratense* and combined percentages of geranial, nerol, and neral (geranial + nerol + neral) in the *T. pulegioides* habitats investigated. Results in natural habitats and under laboratory conditions can differ [21]. Chemical compounds emitted by plants and affected by biotic and abiotic conditions interact with each other in plant communities enabling new chemical compounds to form. For example, monoterpenes can indirectly affect soil micro-organisms inhibiting the nitrification process [50,51].

The laboratory experiment in the present study showed that, though essential oils of both *T. pulegioides* chemotypes were characterized by a phytotoxic effect on *P. pratense*, the essential oil of the geraniol chemotype when compared with the carvacrol chemotype exhibited stronger inhibition of seed germination of this monocotyledon (Table 6). The essential oil of the *T. pulegioides* carvacrol chemotype inhibited seed germination and radicle growth of *P. pratense* through air more strongly than through water. It can be postulated that the impact of the carvacrol chemotype is likely to be stronger during diurnal sunshine. Although the correlational connection between cover-abundance of *H. perforatum* and percentage of geraniol and carvacrol in *T. pulegioides* natural habitats was non-significant, laboratory experimentation showed that the phytotoxic effects of essential oil from the geraniol chemotype and pure geraniol on seed germination of this medicinal plant was very strong: none of the seeds germinated (Table 7). Sometimes inhibitory effects of essential oil from non-phenolic chemotypes are stronger than from phenolic chemotypes. For example, essential oil from *T. pulegioides* geraniol chemotype inhibited seed germination of the monocotyledon *Poa pratensis* through water more strongly than essential oil from the carvacrol chemotype [21]. Interestingly, pure carvacrol stimulated *H. perforatum* seed germination through water with a germination index 1.6 times higher than in control. It can be postulated that under sunny conditions, carvacrol can be inhibitory, but rainy and wet conditions stimulate *H. perforatum* seed germination. Published data also indicate that essential oils can simultaneously exhibit stimulatory effects on other plants [52,53].

Trends in phytotoxic effects of essential oils of carvacrol and geraniol chemotypes and their respective primary compounds, carvacrol and geraniol, did not always coincide. This may be related to synergistic or antagonistic effects of the main compounds interacting with other chemical constituents of these essential oils. Carvacrol and geraniol were rich compounds, however, other chemical constituents in the essential oils comprised significant proportions. For example, the combined percentage of *p*-cymene and γ-terpinene in essential oil from the carvacrol chemotype and combined percentages of geranial, nerol, and neral in essential oil from the geraniol chemotype were 27.85% and 21.54%, respectively (Table 4 and Table 5). Although the bioactive potential of an essential oil usually correlates with the quantity of its main chemical compound [54], this present study demonstrates a possible effect from the quality of the essential oil composition.

Field results showed that carvacrol, the main compound of *T. pulegioides* carvacrol chemotype, is common throughout Lithuania’s territory, whereas the geraniol, the main compound of geraniol chemotype, is predominantly located under the continental climatic conditions. The laboratory experiment demonstrated different phytotoxic effects of essential oil of the geraniol and carvacrol chemotypes: it was established that there was stronger inhibition of *P. pratense* seed germination by the essential oil of the geraniol chemotype than the carvacrol chemotype, and that none of the *H. perforatum* seeds germinated after exposure to the essential oil of the geraniol chemotype. In general, this study builds on previous studies by providing further evidence that different *T. pulegioides* chemotypes have contrasting phytotoxic effects on neighboring plants, including economically valuable forage grasses and pharmacologically valuable medicinal plants, within their natural habitats, affecting the seed germination and/or the seedling development.

## 4. Materials and Methods

### 4.1. Plant Material

In total, 131 different *T. pulegioides* habitats were investigated in Lithuania. These habitats of *T. pulegioides* represented the entire territory of Lithuania and were chosen by random. The distances between habitats were not less than 10 km. A phytocoenological study of each habitat was made in 16 m^2^ fields of meadows according to the methodology of J. Braun-Blanquet [23]. Plant communities were distinguished according to the vegetation classification systems of J. Balevičienė and J. Balevičienė et al. [19,20].

To estimate the distribution of carvacrol and geraniol, the aerial parts of *T. pulegioides* were separately collected from each habitat during the full flowering stage in the following way: the same selected mass of the aerial component of *T. pulegioides* was cut from each individual plant growing in the habitat and pooled. The weight of material sampled depended on each habitat abundance and/or size of individual plants in the habitat: 10 g of the aerial component from each individual plant of *T. pulegioides* was cut in large habitats (where were a lot of individuals), but 30–50 g from each individual was cut in small habitats (where were several individuals). The aerial parts of the plants were dried at room temperature over 4–5 days.

One individual of the *T. pulegioides* carvacrol chemotype and one individual of the *T. pulegioides* geraniol chemotype were transplanted from natural habitat to the field collection of the Nature Research Centre (Vilnius, Lithuania) and vegetatively propagated. The aerial parts of these plants were collected at the full flowering stage and dried at room temperature. Essential oil isolated from these plants was used for phytotoxic analysis.

### 4.2. Isolation and Analysis of Essential Oils

The essential oil was isolated from each mix (as described above, one mix represented the pooled raw material of one *T. pulegioides* habitat) separately using 2 h hydrodistillation with a Clevenger apparatus [22]. For further investigations, 1% solutions of essential oils were prepared in a mixture of diethyl ether and *n*-pentane (1:1). Essential oils analysis was based on a GC-2010 Plus instrument equipped with a GC-QP 2010 Plus (Shimadzu) series mass selective detector in the electron impact ionization mode at 70 eV. Separation of compounds was performed in a fused silica (100% dimethyl polysiloxane) column (30 m × 0.25 mm ID × 0.25 µm film thickness) (Restek, Bellefonte, PA, USA), splitless injection; helium as carrier gas at a flow rate of 1.6 mL/min, injector and detector temperatures 250 °C. GC oven temperature programme: initial temperature of 50 °C (isothermal for 7 min) was increased to 250 °C at the rate of 4 °C/min (isothermal for 5 min) and further increased at the rate of 30 °C/min to 300 °C, and the final temperature was kept for 2 min. Identification of the essential oil compounds was based on a comparison of retention indices (RIs) [24], computer mass spectra library (NBS75K), and analytical standards of α-TA, α-terpinene, *p*-cymene, limonene, γ-terpinene, linalool, nerol, geraniol, β-caryophyllene, caryophyllene oxide, carvacrol, thymol (Sigma-Aldrich, St. Louis, MO, USA). The retention indices were determined relative to the retention times of a series of *n*-alkanes (C7–C30) with linear interpolation. The quantitative analysis was performed using a FOCUS GC (Thermo Scientific, Waltham, MA, USA) gas chromatograph with a flame ionization detector (FID) on a silica capillary column TR-5MS (30 m × 0.25 mm ID × 0.25 μm film thickness) (Thermo Electron Corporation, USA) under the same chromatographic conditions. The percentage amounts of the compounds were recalculated according to the areas of the FID chromatographic peaks assuming that all constituents of the essential oil comprise 100%.

### 4.3. Analysis of Phytotoxic Effect

Seeds of *P. pratense* and *H. perforatum* were bought from JSC Agrofirma “Sėklos” (Lithuania). The effect of essential oils of *T. pulegioides* carvacrol and geraniol chemotypes, and carvacrol and geraniol analytical standards on germination and growth of radicles of *P. pretense* and *H. perforatum* were investigated through air and water. The investigation through air was carried out as follows: filter paper was moistened with 15 mL of distilled water and placed in a Petri dish; small aluminum containers with 3 μL pure essential oil or pure analytical standard were placed in the center of each Petri dish (on the moistened filter paper), thus only aerial contact was allowed between allelochemicals and seeds. In parallel with these experiments were controls with distillate water only. The investigation through water was carried out as follows: filter paper was moistened with 3 μL essential oil or analytical standard dissolved in 1% of Tween 20 and placed in a Petri dish. In total, 100 seeds were overspread gradually in each Petri dish; the borders of the Petri dishes were closed with adhesive tape to preserve the volatile compounds inside and stored at room temperature. In parallel with these experiments were controls with distillate water only. Every treatment was repeated three times. The number of seeds germinating was enumerated. The experiment concluded when the seeds ceased to germinate. The total number of seeds that germinated was determined after the experiment and recalculated to give a final germination percentage (GP). The mean daily germination (MDG) index was calculated from the following equation MDG = GP/d, where GP—the final germination percentage; d—days to the maximum of final germination. The germination index (GI) was calculated as follows: GI = Σ G_t_/T_t_, where G_t_—the number of seeds germinated of day t; T_t_—the number of days at the beginning of the experiment. After the experiment, radicles were measured in each Petri dish (30 radicles per treatment) to establish the radicles length (RL).

### 4.4. Statistical Analysis

Means, standard deviations (SD), min, max values, and coefficients of variation (CV) were used for descriptive statistics of results. The grouping of *T. pulegioides* habitats according to chemotype-determining chemical compounds (carvacrol, thymol, p-cymene, γ-terpinene, geraniol, geranial, nerol, neral, linalool, and α-terpinyl acetate) was performed using the cluster analysis of Ward’s method. The Spearman rank correlation analysis was used to test the correlation of cover-abundance of *P. pratense* with percentages of carvacrol, geraniol, and combined geranial, neral, nerol (geranial + neral + nerol). Additionally, the Spearman rank correlation analysis was used to test correlation of cover-abundance of *H. perforatum* with percentages of carvacrol, geraniol, and combined of geranial, neral, nerol. The Mann–Whitney U test was used to estimate the differences between effects of essential oil and control (and between analytical standard and control) on germination parameters (GP, MGD, GI) of *P. pratense* and *H. perforatum*. Student’s *t* test was used to estimate the differences between effects of essential oil and control (and between analytical standard and control) on radicle development of the same plants. Statistical data processing was carried out with the STATISTICA^®^7 and MS Excel software.

## Figures and Tables

**Table 1:** Distribution of carvacrol and geraniol in *Thymus pulegioides* habitats (N = 131).

Percentages of Carvacrol or Geraniol	Number of Habitats	
	With Carvacrol	With Geraniol
Not found	1	17
(0.00–5.00]	5	67
(5.00–10.00]	20	16
(10.00–15.00]	31	10
(15.00–20.00]	25	8
(20.00–25.00]	23	5
(25.00–30.00]	12	3
(30.00–35.00]	8	3
(35.00–40.00]	2	2
(40.00–45.00]	3	0
(45.00–50.00]	1	0

**Table 2:** Descriptive statistics of percentages of carvacrol, thymol, geraniol, linalool, and α-terpinyl acetate chemotype-determining main chemical compounds in distinguished clusters of *Thymus pulegioides* habitats and habitats nos. 11, 34, 39, 99, 100, and 106 (SD—standard deviation; Min—minimum value; Max—maximum value; CV—coefficient of variation).

Cluster or Habitat Number		Percentage of Compound									
		Carvacrol	Thymol	p-Cymene	γ-Terpinene	Geraniol	Geranial	Nerol	Neral	Linalool	α-Terpinyl Acetate
Cluster 1′ (N = 34)	Mean	11.24	1.55	4.94	8.86	18.56	3.09	9.92	8.63	2.05	4.94
	SD	5.51	2.46	3.85	6.36	8.90	1.44	5.40	6.18	4.94	2.01
	Min	0.00	0.00	0.39	0.00	3.48	0.32	0.88	0.65	0.00	0.00
	Max	24.28	10.28	12.60	23.85	39.87	6.57	20.57	34.92	22.94	9.84
	CV, %	49	159	78	72	48	47	54	72	241	41
Cluster 2′ (N = 17)	Mean	13.38	12.10	17.61	16.59	2.39	0.40	1.43	1.02	0.36	0.27
	SD	4.12	7.48	7.27	6.59	2.85	0.46	1.73	1.35	0.10	0.53
	Min	7.09	4.50	5.20	0.81	0.00	0.00	0.00	0.00	0.16	0.00
	Max	21.31	31.00	29.55	30.64	10.04	1.45	5.66	4.23	0.51	1.84
	CV, %	31	62	41	40	119	115	121	132	28	196
Cluster 3′ (N = 45)	Mean	26.11	2.09	9.44	23.78	1.89	0.29	0.75	0.56	0.56	0.07
	SD	8.75	2.96	4.41	6.40	2.21	0.40	1.13	0.90	0.75	0.24
	Min	12.10	0.00	1.23	11.96	0.00	0.00	0.00	0.00	0.00	0.00
	Max	48.00	11.11	20.00	42.60	7.80	1.40	5.29	4.16	4.41	1.35
	CV, %	34	142	47	27	117	138	151	161	134	343
Cluster 4′ (N = 28)	Mean	17.89	1.56	20.66	17.49	2.94	0.72	2.72	2.06	0.35	0.34
	SD	5.38	1.72	7.17	5.96	2.66	0.60	2.59	1.97	0.22	1.01
	Min	8.19	0.00	5.73	4.40	0.00	0.00	0.00	0.00	0.10	0.00
	Max	27.10	6.36	38.49	29.47	10.04	1.88	9.34	7.18	1.23	4.33
	CV, %	30	111	35	34	90	83	95	96	63	297
no. 11		13.58	2.38	20.99	11.68	1.67	0.17	0.65	0.09	14.25	2.34
no. 34		5.65	1.32	6.82	5.87	2.92	0.56	1.87	1.43	0.35	43.56
no. 39		5.65	0.00	20.70	6.44	2.08	0.12	0.07	0.00	6.51	30.77
no. 99		0.06	0.00	0.14	0.48	12.35	1.12	3.52	2.81	0.29	57.50
no. 100		3.48	0.27	1.31	1.57	4.01	0.38	1.01	0.90	57.75	6.01
no. 106		6.39	0.00	5.38	5.85	6.36	1.32	4.78	3.87	40.37	3.99

**Table 3:** Distribution of species of medicinal plants, raw materials of which are included in the European Pharmacopoeia, and characteristic Poaceae species of phytocoenological vegetation classes *Molinio-Arrhenatheretea elatiori*, *Trifolio-Geranietea sanguinei*, *Koelerio-Corynephoretea,* and *Festuco-Brometea erecti* in investigated *Thymus pulegioides* habitats.

Species	Number of Investigated Habitats with Different Cover-Abundances ^1^					Percentage of Habitats, Where This Species Was Found, %
	+	1	2	3	4	
Species of medicinal plants						
*Achillea millefolium* L.	70	17	1	–	–	67
*Arrhenatherum elatius* (L.) P. Beauv. Ex J. Presl et C. Presl	–	1	–	–	–	1
*Agrimonia eupatoria* L.	27	12	3	–	–	32
*Alchemilla* sp.	19	1	–	–	–	20
*Artemisia absinthium* L.	1	–	–	–	–	1
*Betula pendula* Roth	22	2	–	–	–	18
*Carum carvi* L.	3	–	–	–	–	2
*Centaurium erythraea* Rafn	4	1	–	–	–	4
*Chelidonium majus* L.	1	–	–	–	–	1
*Elytrigia repens* (L.) Nevski	8	1	–	–	–	7
*Equisetum arvense* L.	33	13	2	3	–	39
*Fagopyrum esculentum* Moench	1	–	–	–	–	1
*Filipendula ulmaria* (L.) Maxim.	1	–	–	–	–	1
*Frangula alnus* Mill.	5	–	–	–	–	4
*Fumaria officinalis* L.	1	–	–	–	–	1
*Hypericum perforatum* L.	49	–	–	–	–	37
*Juniperus communis* L.	2	–	–	–	–	2
*Melilotus officinalis* (L.) Pall.	1	–	–	–	–	1
*Mentha arvensis* L.	2	–	–	–	–	2
*Oenothera biennis* L.	13	1	–	1	–	12
*Origanum vulgare* L.	3	–	–	3	–	5
*Pinus sylvestris* L.	32	6	1	–	–	30
*Plantago lanceolate* L.	23	11	2	–	–	28
*Potentilla erecta* (L.) Raeuschel	3	–	–	–	–	2
*Primula veris* L.	8	2	–	–	–	8
*Prunella vulgaris* L.	27	1	–	–	–	21
*Quercus robur* L.	18	–	–	–	–	14
*Ribes nigrum* L.	1	–	–	–	–	1
*Rubus idaeus* L.	6	–	–	–	–	5
*Solidago virgaurea* L.	17	3	–	–	–	15
*Salix purpurea* L.	3	–	–	–	–	2
*Taraxacum officinale* F. H. Wigg.	18	16	2	2	–	29
*Tilia cordata* Mill.	2	–	1	1	–	3
*Urtica dioica* L.	2	–	–	–	–	2
*Valeriana officinalis* L.	2	–	–	–	–	2
Species of genus Poaceae						
*Dactylis glomerata* L.	37	41	13	3	1	73
*Deschampsia cespitosa* (L.) P. Beauv.	6	1	–	–	–	5
*Festuca ovina*-L.	10	–	–	–	–	8
*Festuca pratensis* Huds.	15	14	4	1	–	26
*Festuca rubra* L.	4	20	8	1	–	25
*Lolium perrene* L.	4	1	–	–	–	4
*Phleum pratense* L.	34	6	1	–	–	31
*Poa annua* L.	1	2	2	1	–	5
*Poa pratensis* L.	5	15	10	3	2	27
*Poa trivialis* L.	1	–	–	–	–	1

^1^ Cover-abundance of species determined according to Braun-Blanquet [23].

**Table 4:** Composition of essential oil of the *Thymus pulegioides* carvacrol chemotype (GC area %; RI—retention index; MS—mass spectrum; Std—analytical standard). Mass spectral similarities of investigated compounds were 85–96% (in comparison with computer mass spectra library (NBS75 K) and/or analytical standards).

Compound	Identification Method	Retention Index		GC Area, %
		Calculated	Literature [24]	
α-Thujene	RI, MS	932	924	1.14
α-Pinene	RI, MS	940	932	0.54
1-octen-3-ol	RI, MS	979	971	1.79
α-Terpinene	RI, MS, Std	1022	1014	2.01
p-Cymene	RI, MS, Std	1029	1020	12.38
Limonene	RI, MS, Std	1032	1024	0.40
(E)-β-Ocimene	RI, MS	1042	1044	0.40
γ-Terpinene	RI, MS, Std	1053	1054	15.47
Borneol	RI, MS, Std	1173	1165	0.42
Terpinen-4-ol	RI, MS, Std	1172	1174	0.34
α-Terpineol	RI, MS	1194	1186	0.09
Nerol	RI, MS, Std	1235	1227	0.47
Neral	RI, MS,	1242	1235	0.17
Geraniol	RI, MS, Std	1237	1249	2.31
Geranial	RI, MS,	1272	1264	0.16
Thymol	RI, MS, Std	1298	1289	0.29
Carvacrol	RI, MS, Std	1308	1298	23.71
Neryl acetate	RI, MS	1368	1359	0.07
β-Bourbonene	RI, MS	1395	1387	0.21
β-Caryophyllene	RI, MS, Std	1426	1417	6.87
α-Humulene	RI, MS	1460	1452	0.06
cis-β-Guaiene	RI, MS	1500	1492	1.45
β-Bisabolene	RI, MS	1513	1505	3.36
(E)-β-Farnesene	RI, MS	1463	1454	0.26
(E)-β-Ionone	RI, MS	1496	1487	0.15
Caryophyllene oxide	RI, MS, Std	1591	1582	1.77
Monoterpene hydrocarbons				32.34
Oxygenated monoterpenes				42.16
Sesquiterpene hydrocarbons				12.21
Oxygenated sesquiterpenes				1.38
Other				2.01
Total identified				90.10

**Table 5:** Composition of essential oil of the *Thymus pulegioides* geraniol chemotype (GC area %; RI—retention index; MS—mass spectrum; Std—analytical standard). Mass spectral similarities of investigated compounds were 85–96% (in comparison with computer mass spectra library (NBS75 K) and/or analytical standards).

Compound	Identification Method	Retention Index		GC Area, %
		Calculated	Literature [24]	
α-Pinene	RI, MS	941	932	0.29
1-octen-3-ol	RI, MS	980	971	0.61
α-Terpinene	RI, MS, Std	1023	1014	0.02
p-Cymene	RI, MS, Std	1029	1020	0.17
Limonene	RI, MS, Std	1033	1024	0.13
(E)-β-Ocimene	RI, MS	1053	1044	0.05
γ-Terpinene	RI, MS, Std	1063	1054	0.49
Linalool	RI, MS, Std	1104	1095	0.71
Nerol oxide	RI, MS	1163	1154	0.16
Borneol	RI, MS, Std	1174	1165	0.82
Terpinen-4-ol	RI, MS, Std	1183	1174	0.17
α-Terpineol	RI, MS	1195	1186	0.08
Nerol	RI, MS, Std	1236	1227	9.99
Neral	RI, MS,	1245	1235	7.68
Geraniol	RI, MS, Std	1260	1249	55.99
Geranial	RI, MS,	1274	1264	3.87
α-Terpinyl acetate	RI, MS, Std	1355	1346	0.14
Neryl acetate	RI, MS	1368	1359	1.75
β-Bourbonene	RI, MS	1396	1387	0.21
β-Caryophyllene	RI, MS, Std	1426	1417	6.67
cis-β-Guaiene	RI, MS	1501	1492	1.21
β-Bisabolene	RI, MS	1514	1505	1.04
(E)-β-Farnesene	RI, MS	1463	1454	0.26
Caryophyllene oxide	RI, MS, Std	1491	1582	1.77
Monoterpene hydrocarbons				1.15
Oxygenated monoterpenes				79.58
Sesquiterpene hydrocarbons				9.39
Oxygenated sesquiterpenes				1.77
Other				2.52
Total identified				94.59

**Table 6:** Phytotoxic effect of essential oils of *Thymus pulegioides* carvacrol, geraniol chemotypes, and analytical standards of carvacrol, geraniol through air and water on *Phleum pratense* (GP—germination percentage; MDG—mean daily germination; GI—germination index; SD—standard deviation). Letters denote statistically significant (*p* < 0.05) differences: lower letters between essential oil and control, capital letters between analytical standard and control.

Effect	Chemical		GP ± SD, %	MGD, %	GI, Seeds/Day	Radicle Development ± SD, mm
Control			96.67 ± 0.50	12.13	31.38	9.77 ± 4.29
Through air	Essential oil	Geraniol chemotype	1.25 ± 0.96 ^a^	0.07 ^a^	0.08 ^a^	5.16 ± 5.57
		Carvacrol chemotype	11.20 ± 3.83 ^a^	0.95 ^a^	2.11 ^a^	2.13 ± 3.87 ^a^
	Analytical standard	Geraniol	10.25 ± 3.86 ^A^	0.97 ^A^	1.60 ^A^	1.77 ± 0.66 ^A^
		Carvacrol	18.00 ± 3.81 ^A^	2.94 ^A^	4.87 ^A^	1.40 ± 0.75 ^A^
Through water	Essential oil	Geraniol chemotype	47.00 ± 7.87 ^a^	2.6 ^a^	4.84 ^a^	7.00 ± 5.22
		Carvacrol chemotype	92.00 ± 1.41 ^a^	8.09 ^a^	20.32 ^a^	5.69 ± 4.89 ^a^
	Analytical standard	Geraniol	1.80 ± 1.79 ^A^	0.18 ^A^	0.47 ^A^	1.22 ± 0.75 ^A^
		Carvacrol	88.33 ± 10.70 ^A^	7.95 ^A^	17.48 ^A^	4.38 ± 3.43 ^A^

**Table 7:** Phytotoxic effect of essential oils of *Thymus pulegioides* carvacrol, geraniol chemotypes, and analytical standards of carvacrol, geraniol through air and water *Hypericum perforatum* (GP—germination percentage; MDG—mean daily germination; GI—germination index; SD—standard deviation). Letters denote statistically significant (*p* < 0.05) differences: lower letters between essential oil and control, capital letters between analytical standard and control.

Effect	Chemical		GP ± SD, %	MGD, %	GI, Seeds/Day	Radicle Development ± SD, mm
Control			46.67 ± 7.02	2.59	4.94	3.31 ± 1.24
Through air	Essential oil	Geraniol chemotype	0.00 ^b^	0.00 ^b^	0.00 ^b^	–
		Carvacrol chemotype	28.00 ± 6.09 ^b^	1.98 ^b^	3.45 ^b^	1.63 ± 0.51 ^b^
	Analytical standard	Geraniol	0.00 ^B^	0.00 ^B^	0.00 ^B^	–
		Carvacrol	19.67 ± 4.62 ^B^	1.20 ^B^	1.74 ^B^	0.87 ± 0.59 ^B^
Through water	Essential oil	Geraniol chemotype	0.00 ^b^	0.00 ^b^	0.00 ^b^	–
		Carvacrol chemotype	37.60 ± 9.84	2.42	5.34	1.75 ± 0.76 ^b^
	Analytical standard	Geraniol	0.00 ^B^	0.00 ^B^	0.00 ^B^	–
		Carvacrol	55.00 ± 7.00	3.45	7.70 ^B^	1.60 ± 0.59 ^B^

## Data Availability

The data presented in this study are available in Supplementary Material.

## Supplementary Materials

Supplement S1: Two dimensional dendrogram of habitats of Thymus pulegioides performed on the basis of chemotypes determining chemical compounds.
