# Supplementary material for: Geraniol and Carvacrol in Essential Oil Bearing Thymus pulegioides: Distribution in Natural Habitats and Phytotoxic Effect
Source: Molecules. 2022 Feb 1;27(3):986. doi: 10.3390/molecules27030986 (PMC8838350; doi:10.3390/molecules27030986)
Supplement: Supplementary file 1 [file molecules-27-00986-s001.zip › molecules-1565730-supplementary.pdf]

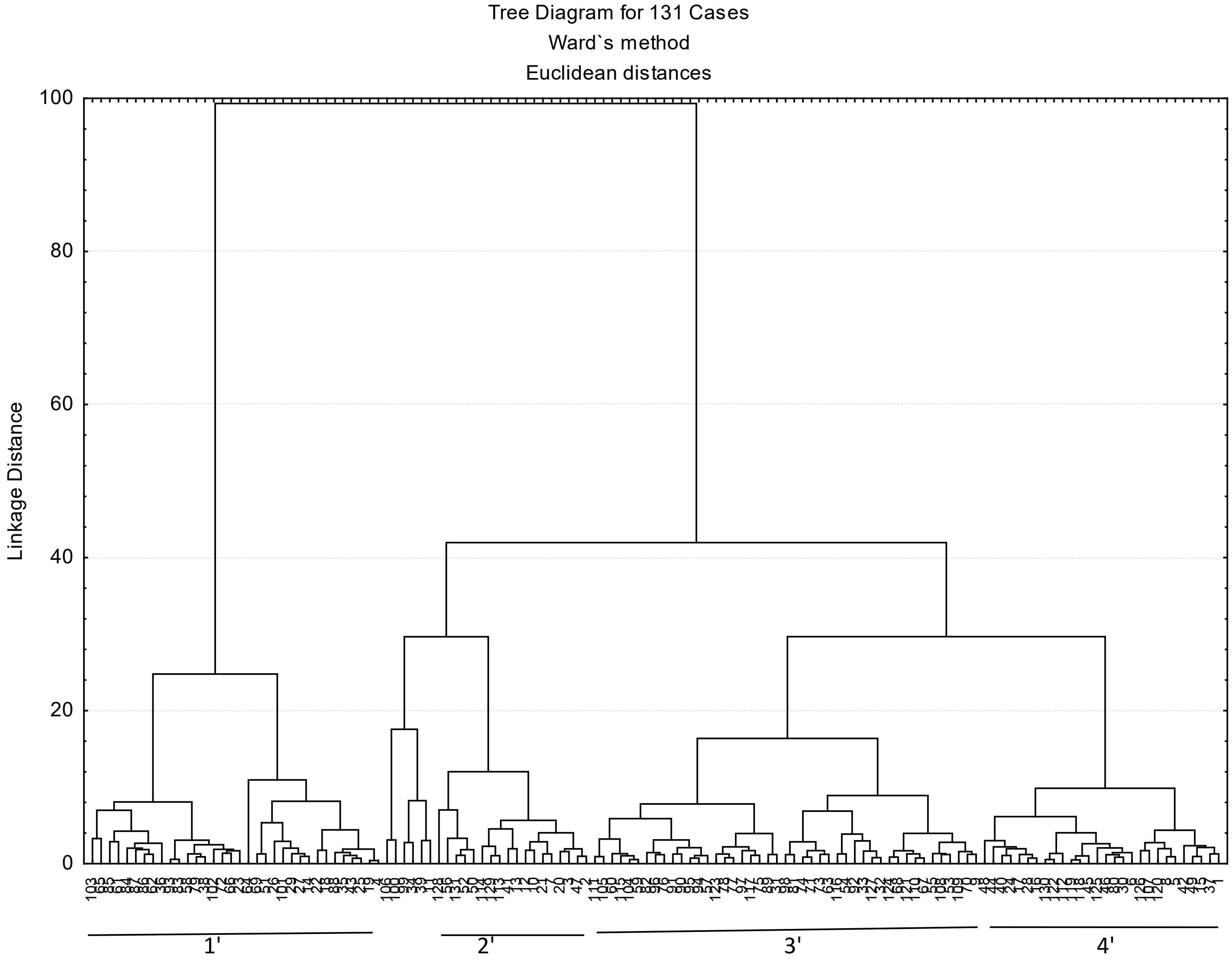

Supplement S1. Two dimensional dendrogram of habitats of *Thymus pulegioides* performed on the basis of chemotypes determining chemical compounds.
